# Sagittal Craniosynostosis with Uncommon Anatomical Pathologies in a 56-Year-Old Male Cadaver

**DOI:** 10.1155/2019/8034021

**Published:** 2019-12-08

**Authors:** Andrey Frolov, Craig Lawson, Joshua Olatunde, James T. Goodrich, John R. Martin III

**Affiliations:** ^1^Center for Anatomical Science and Education, Department of Surgery, Saint Louis University School of Medicine, Saint Louis, MO 63104, USA; ^2^Departments of Neurological Surgery, Pediatrics, Plastic and Reconstructive Surgery, Albert Einstein College of Medicine, Montefiore Medical Center, Bronx, NY 10467, USA

## Abstract

Sagittal craniosynostosis (CS) is a pathologic condition that results in premature fusion of the sagittal suture, restricting the transverse growth of the skull leading in some cases to elevated intracranial pressure and neurodevelopmental delay. There is still much to be learned about the etiology of CS. Here, we report a case of 56-year-old male cadaver that we describe as sagittal CS with torus palatinus being an additional anomaly. The craniotomy was unsuccessful (cephalic index, CI = 56) and resulted in abnormal vertical outgrowth of the craniotomized bone strip. The histological analysis of the latter revealed atypical, noncompensatory massive bone overproduction. Exome sequencing of DNA extracted from the cadaveric tissue specimen performed on the Next Generation Sequencing (NGS) platform yielded 81 genetic variants identified as pathologic. Nine of those variants could be directly linked to CS with five of them targeting RhoA GTPase signaling, with a potential to make it sustained in nature. The latter could trigger upregulated calvarial osteogenesis leading to premature suture fusion, skull bone thickening, and craniotomized bone strip outgrowth observed in the present case.

## 1. Introduction

CS is a condition that affects ~1 in 2,000–2,500 newborns and manifests itself as a premature fusion of a single or multiple cranial suture(s) leading to the deformation of a skull shape [1–3]. The latter occurs due to a restriction of the skull growth in the direction perpendicular to the fused suture. Based on the etiology, CS can be classified as either primary or secondary. The former occurs as a result of genetic, environmental, or a combination of thereof factors specifically targeting cranial sutures without causing a major pathological impact on the rest of the human body. Secondary CS develops as a result of mechanical impacts, metabolic disorders such as hyperthyroidism, hypercalcemia, vitamin D deficiency etc. that targets cranial sutures nonspecifically, or due to premature suture closure as a result of the impaired developmental program that regulates brain growth [4]. In turn, primary CS can occur as an isolated event resulting in nonsyndromic CS, or it may less frequently be associated with other anomalies leading to syndromic CS [1–3]. Despite a significant progress made in recent years, there is still much to be learned regarding the etiology of CS, particularly its genetic underlining [3].

Therefore, the main objectives of this study were to: (i) characterize the craniofacial pathology (scaphocephaly) observed in the 56-year-old cadaver and (ii) gain insights into its genetic component by identifying the respective genetic variants through exome sequencing of DNA extracted from tissue procured from the donor's body. A clearer understanding of the nature of the above pathology may help to better delineate the mechanism(s) responsible for its development, as a well as may improve outcomes of the specialized corrective clinical procedures.

## 2. Case Presentation

### 2.1. Anatomical Characterization

#### 2.1.1. Human Cadaveric Body Procurement

A 56-year-old male cadaver was received through Saint Louis University (SLU) School of Medicine Gift of Body Program from an individual who had given his written informed consent. The available medical record indicated that this individual had a history of moderate mental retardation, cerebral palsy, seizure disorder, scoliosis, hydrocephalus, joint pain, mood disorder, anxiety disorder, encephalopathy and leukopenia. The cause of death was indicated as cerebral palsy. The cadaveric head was separated from the extremely contracted body and embalmed using 2 : 1 mixture of ethylene glycol and isopropyl alcohol.

#### 2.1.2. CT Imaging

**Figure 1:**
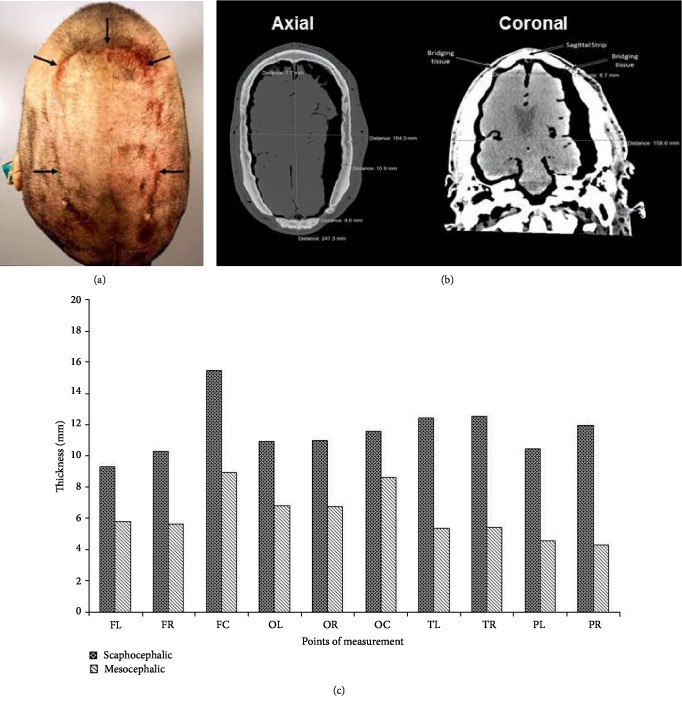
(a) Physical examination of the scaphocephalic cadaver head. Superior view shows the demarcation of the displaced sagittal strip (black arrows). (b) Computed Tomography (CT) images of the cadaver head. Left: The axial view reveals a thickened skull and spaces of bone towards the posterior aspect of the skull. The long, narrow skull yielded a cranial vault index of 0.56. The brain appears to have undergone significant atrophy. Right: The coronal view shows an abnormal thinning of the skull on each side of the sagittal suture near the superior aspect of the skull. These areas likely coincide with the areas lacking bone in the axial view. (c) Increased bone thickness in the scaphocephalic skull of the individual with CS. The thickness of the frontal, parietal, occipital and temporal bones was measured in five male mesocephalic skulls (normal, dark grey) at the bony points described in [5] using Neiko digital calipers. The measurements of the frontal bones were conducted approximately 15 mm above the supraorbital ridges at three points: center point (FC) and 2 cm away from the center point on the left (FL) and right (FR). The occipital bones were measured approximately 4 cm above the external occipital protuberance at three points: center point (OC) and 2 cm away from the center point on the left (OL) and right (OR). Thickness of the temporal bones were measured at the level of the zygomaticofrontal suture on the left (TL) and right (TR) sides. The parietal bones were measured approximately 1 cm above the most superior point of the squamosal suture on the left (PL) and right (PR) side. The same measurements were conducted on the scaphocephalic cadaveric head (CS, pattern) at the bony points described above using Syngo Fast-View software. Data shown are mean of three measurements for a single scaphocephalic skull and 15 measurements for five mesocephalic skulls (three measurements per skull).

The initial visual examination of the embalmed patient's head revealed its abnormal, scaphocephalic, shape as well as a presence of bulging sagittal bone strip (Figure 1(a)). The subsequent CT image analysis confirmed the scaphocephaly (CI = 56) and demonstrated clearly a significant bone thickening in the scaphocephalic skull as compared to mesocephalic skulls (Figure 1(b)). The respective fold change varied from 1.34 for occipital bone to 2.76 for parietal bone with the rest of the scaphocephalic skull bone thickening falling into the ~1.6–2.3 fold range (Figure 1(c)). It should be noted, that the bone thickness values derived in the current report from five mesocephalic skulls (Figure 1(c)) could be viewed as a representative snapshot of a large respective sampling because they were very similar to those reported for the group of 66 male mesocephalic skulls [5].

#### 2.1.3. Craniectomy

**Figure 2:**
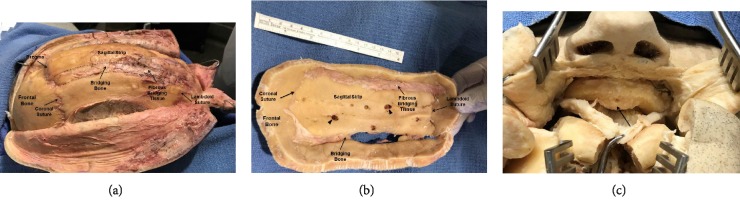
Examination of the exposed calvarium. (a) The exposed calvarium shows the presence of the coronal and lambdoid sutures. The vertical displacement of the sagittal strip is apparent at bregma. At the sagittal strip—parietal bone junction (dashed lines), there are areas of bridging bone and fibrous bridging tissue. (b) Internal view of the calvarium. Black arrowheads—large arachnoid granulations. (c) Torus palatinus is evident in the midline (arrow) with the epithelium reflected.

Upon closer examination of the individual's head it was concluded that he underwent, most likely early in infancy, a neurosurgical procedure, a sagittal strip craniotomy, with a likely effort to correct the anomalous skull shape and to reduce intracranial pressure. One of the most interesting features of the present case is an abnormal re-growth of the surgically removed bone strip and the resultant elevated vertical displacement of the skull (Figure 2(a)). It appears that the oval segment in question was resected and then replaced *in situ* without fixation or stabilization, thereby permitting some adjustment of the calvarial vault and potentially lowering the intracranial pressure from the underlying cerebral hemispheres. Examination of the calvarial region revealed an oval segment of calvarial bone that included remnants of frontal and parietal bone with all suture lines obliterated (Figure 2(b)). This material was separated from the surrounding calvarium by a variable band of grossly fibrous tissue that was adherent to edges of the original cranial bones and which was also adherent to the underlying dura. At a few locations there was a confluence of healed bone between the original, surgically created margins and the oval bone segment removed and replaced at the time of craniotomy (Figure 2(b)).

Also importantly, examination of the dural surface of the calvarium revealed several deep granular foveolae indicative of large arachnoid granulations in the sagittal strip (Figure 2(b)) that are likely to be the result of increased intracranial pressure.

#### 2.1.4. Mandibulotomy

Physical examination of the maxillofacial features of the cadaveric head revealed a large underbite that prompted the dissection of the mandible to probe for additional abnormalities. The mandible was exposed by removing the soft tissue from the mental surface followed by bisection of the bone and tongue. This procedure revealed an exostotic hard palate (torus palatinus) and complete edentulism (Figure 2(c)).

### 2.2. Histological Analysis

**Figure 3:**
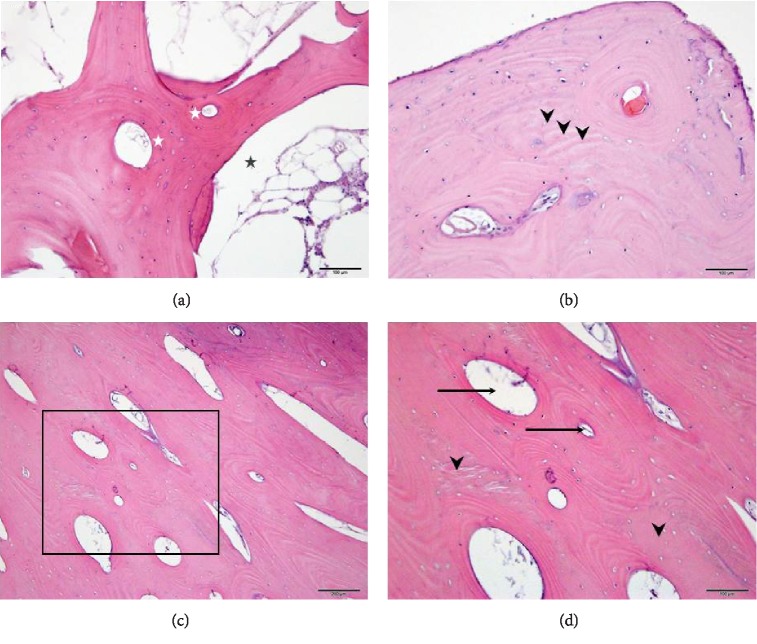
Histological analysis of the scaphocephalic calvarium. (a) The sagittal strip displays cancellous bone with variably sized osteons (white stars). Intervening medullary spaces (black star) contain typical myeloid cellular elements, but without the presence of osteoclasts. (b) The bridging bone demonstrates scattered immature Haversian systems. Areas suggestive of osteon remnants are indicated by the arrowheads. (c) An additional image through the bridging bone shows dense, confluent areas of well-formed Haversian systems characteristic of typically formed compact or cortical bone. (d) Enlarged boxed area in C shows variably sized Haversian systems of similar orientation (arrows). Portions of the image indicated by arrowheads suggest immature (woven bone) that has been replaced by newer Haversian systems resulting in the formation of compact bone.

Sections of bony tissue from the sagittal strip revealed areas of immature compact bone with incomplete or developing Haversian systems, whose orientation was predominately perpendicular to the section orientation (Figure 3(a)). Areas of immature bone were located on either side of randomly oriented bony spicules with marrow spaces among them. These marrow areas contained small foci of both red and white cell precursors, with larger numbers of unilocular adipocytes.

More importantly, sections of bony tissue from surgically created margins revealed an extremely high number of osteons, with some, well-formed and others, formed incompletely (Figures 3(b)–3(d)). In some areas, there was a lack of clear cement lines and there were also no osteoclasts or Howship lacunae present, nor were there any evidence of diploe. The lack of cement lines, osteoclasts and diploe, along with the high number of osteons would be consistent with a massive atypical bone overproduction without adequate compensatory bone degradation thereby leading to much thicker skull bone formation.

### 2.3. Genetic Analysis

The genetic underlining of the present case was addressed by performing a genetic screen for the putative variants using NGS technology applied to DNA extracted from the respective cadaveric tissue specimen as described previously [6, 7]. Additional experimental details pertinent to the performed bioinformatics analysis are provided in [Supplementary-material supplementary-material-1].

The sequencing of the DNA coding regions (exome) yielded 81 rare genetic variants (minor allele frequency, MAF ≤0.01) with predicted deleterious (pathological) implications ([Supplementary-material supplementary-material-1]). Nine of those variants could be linked to the CS development (Table 1) with the majority (five) targeting RhoA GTPase activation either directly through *ARHGAP21* and *GMIP* or indirectly through noncanonical Wnt signaling (*INADL* & *RNF213*) and/or *PIEZO1* pathways (Table 1). The remaining variants are those involved in the regulation of osteogenesis/teeth development (*BMP6*) and cilia function (*CEP162*, *CROCC* & *DNAH11*) (Table 1). It should be noted that all nine variants are novel as they have never been reported in association with CS.

## 3. Discussion

The present case of craniofacial malformation could be described as a single suture sagittal CS with the additional associated anatomical pathologies being torus palatinus and complete edentulism. This conclusion was made based on the measured CI of 56 (75–90 being normal) derived from the respective CT images and the mandibulotomy results (Figures 1 and 2). It should be noted, that because without a detailed medical history it is impossible to say when and how the edentulism developed and progressed, the extent of its association with the present case remains uncertain and will not be discussed further. However, the detected *BMP6* genetic variant (Table 1) could be of interest, since while being apparently dispensable for the general osteogenesis [8], *BMP6 *has been reported to positively regulate teeth development in mice and fishes [9, 10].

The uniqueness and importance of this case is several-fold. *First*, this is the only, to the best of our knowledge, reported case of a single suture sagittal CS manifested with torus palatinus. Despite the relatively high prevalence of the latter in the general population, ~26% (average from 15 studies reviewed in [11]), there is almost no information on its manifestation in CS: a single report found in the literature describes its presence in Muenke Syndrome form of coronal CS with a low, 5% incidence [12].

*Second*, it presents a rare opportunity to evaluate the long term results (>50 years) of the corrective surgical procedure for CS which in the current case was, most likely, the sagittal strip craniotomy apparently performed without removal of the frontal bone and its reshaping to correct for the frontal bossing [13, 14]. It is clear that the above procedure was unsuccessful as evidenced by a failure to restore a normal CI value as well as by the abnormal outgrowth of the craniotomized sagittal bone strip. The performed surgical procedure was also unsuccessful if its sole purpose was to relieve an elevated intracranial pressure, which was reported to be present in 10–15% of children with the single suture CS [15, 16]. This conclusion is supported by an appearance of large arachnoid granulations on the dural surface of the sagittal strip (Figure 2(b)) that are most likely caused by the dura pressing against the calvarial bone in response to increased intracranial pressure.

*Third*, the current case provides unique insights into the process of calvarial bone repair/regeneration following cranial trauma in humans. Indeed, as it has been recently stated in [17]: “Compared with long bone fractures, our knowledge of the molecular physiology of healing craniofacial fractures is extremely sparse”. In this regard, the sagittal strip craniotomy, which was most likely performed in the present case and where the resected bone strip was replaced in situ resembles, in general, the autologous bone cranioplasty following decompressing craniectomy [18]. One of the notable complications of the cranioplasty with autologous bone is the bone resorption [19, 20] with the incidence reaching as high as ~62% for the skull defect area in the range of 75–99 cm^2^[18]. It should be noted, that the estimated craniotomized bone strip area of ~89 cm^2^ in the present case (Figure 2(b)) was within that range but no bone resorption was detected (Figure 3).

The bone regeneration represents a delicate balance between the formation of new bone and its resorption. The former process is regulated by the recruitment of osteoblasts to the site of injury and their ossification while the latter process is controlled by the osteoclasts recruitment to and their activity in the bony lesion [21–23]. The normal calvarial bone repair process is accomplished when the newly formed bony tissue assumes the morphology of the original one including the presence of well-developed Haversian systems and diploe, as well as normal osteoclasts count/activity and the bone thickness [22, 24, 25]. None of the above criteria for the normal bone repair/regeneration was fulfilled in the current case. The respective histological data (Figure 3) point toward massive bone overproduction, as evidenced by the extremely high number of osteons, which was not compensated by the bone resorption most likely due to the absence of identifiable osteoclasts in the newly formed bridging bone (Figures 2 and 3). Yet the Haversian systems were immature and there was no evidence of diploe (Figure 3). The abovementioned bone overproduction apparently resulted in the significant outgrowth of the craniotomized sagittal bone strip (Figure 1(a)). None of the histomorphological features described in the current case was reported in the literature either as the complications or the normal outcomes of the cranioplasty in CS [18–20, 26] and, thus, could be considered as unique.

*Fourth,* the results of the genetic screen (Table 1) could provide an important mechanistic insight into the massive bone overproduction described above. RhoA GTPase is a known master regulator of osteogenesis and its sustained activation is required for the initiation of this program [27–29]. In the present case, the sustained RhoA activation can be achieved through three major mechanisms either separately or in any combination thereof: (i) directly, due to mutations in the negative RhoA activity regulators, *ARHGAP21* and *GMIP* (Table 1) [30, 31]; (ii) indirectly, owing to a sustained noncanonical Wnt signaling [32] because of the mutations in the respective negative regulators, *INADL *and* RNF213* (Table 1) [33, 34], and (iii) indirectly, following the sustained mechanosensor PIEZO1 activation as a result of the gain-of-function mutation p.Pro2510Leu (Table 1) [35–37]. Additionally, the PIEZO1 sustained activation could be driven by potentially high scaphocephalic intracranial pressure with the latter also serving as a trigger for the epileptic seizures noted in the medical history of this individual. Therefore, the respective data could identify a novel, RhoA signaling nodule, as a convergence point for several signaling pathways noted above and whose sustained activation might serve as a driving force for the development of CS as well as for the massive, non-compensatory bone overproduction noted in the present case. Yet, the *CEP162*, *CROCC,* and *DNAH11* genetic variants affecting cilia function (Table 1) [38–40] could also contribute to the aberrant osteogenic program in the examined body [41].

It should be noted, that the genetic variants described above, although being identified as deleterious/pathologic by their stringent filtering through the three specific databases [7], can only be viewed as predicted or potentially pathologic in the current case of CS because they were detected by the exome sequencing of a single proband. The studies involving available clinical genetics data are being planned to address this limitation.

*Fifth, *the upregulated osteogenic program due to potentially sustained RhoA signaling described in the present report should be taken into consideration while trying to understand the nature of CS signs and symptoms recurrence in patients following a corrective surgery and who were tested negative for the mutations commonly associated with CS [42–44]. In the latter case, when there is the genetic and/or biochemical evidence pointing toward aberrantly stimulated RhoA signaling, supplementing a surgical procedure with the respective therapeutic treatment(s) aiming to curb an excessive RhoA signaling in the calvarium could provide better clinical outcomes.

*Finally*, this case has a high educational value because it demonstrates clearly that a simple craniotomy of the sagittal suture without additional procedures aiming to reshape the surrounding cranial bones is not going to produce desirable outcomes of this corrective procedure for sagittal CS.

## 4. Conclusion

The current case provides a unique description of the histopathological features following craniotomy of the sagittal bone strip in CS as well as important information pointing toward a potential role of sustained RhoA signaling in the development and progression of sagittal CS.

## Figures and Tables

**Table 1:** Selected deleterious (pathologic) genetic variants associated with the current case of sagittal craniosynostosis.

Gene	Protein function	Variant	MAF
*ARHGAP21*	Rho GTPase Activating Protein 21. Functions as a GTPase-activating protein (GAP) for RHOA and CDC42.	p.Arg492Gly	0.0021
*BMP6*	Bone morphogenetic protein 6. Teeth development. Cartilage development. Endochondral ossification. Positive regulation of osteoblast differentiation. Positive regulation of bone mineralization. Positive regulation of chondrocyte differentiation.	p.Pro93Ser	0.0001
*CEP162*	Centrosomal protein of 162 kDa. Required to promote assembly of the transition zone in primary cilia. Acts by specifically recognizing and binding the axonemal microtubule. Required to mediate CEP290 association with microtubules.	p.Arg802Trp	0.0001
		p.Arg878Trp	
*CROCC*	Rootletin. Major structural component of the ciliary rootlet, a cytoskeletal-like structure in ciliated cells which originates from the basal body at the proximal end of a cilium and extends proximally toward the cell nucleus (by similarity). Required for the correct positioning of the cilium basal body relative to the cell nucleus, to allow for ciliogenesis.	p.Arg637Trp	0.0001
*DNAH11*	Dynein heavy chain 11, axonemal. Force generating protein of respiratory cilia. Produces force towards the minus ends of microtubules. Dynein has ATPase activity; the force-producing power stroke is thought to occur on release of ADP.	p.Pro2006Leu	0.0001
*GMIP*	GEM-interacting protein. Stimulates, in vitro and in vivo, the GTPase activity of RhoA.	p.Pro532Leu	0.0001
		p.Pro535Leu	
		p.Pro561Leu	
*INADL*	InaD-like protein also known as PATJ. Negative regulator of Wnt signaling. Blocks DFz1 activity in the planar cell polarity pathway (PCP) in cooperation with atypical PKC. Fzd/PCP pathway represents the noncanonical Wnt signaling.	p.Glu1499Lys	0.0099
*PIEZO1*	Piezo-type mechanosensitive ion channel component 1. Pore-forming subunit of a mechanosensitive nonspecific cation channel. Plays a key role in osteogenesis. Its activation commits mesenchymal stem cells to osteogenic differentiation.	p.Pro2510Leu	0.0042
*RNF213*	E3 ubiquitin-protein ligase RNF213. Involved in the noncanonical Wnt signaling pathway in vascular development: acts by mediating ubiquitination and degradation of FLNA and NFATC2 downstream of RSPO3, leading to the inhibition of the noncanonical Wnt signaling pathway and promoting vessel regression.	p.Trp4677Leu	0.01

^∗^Variant column describes deleterious (pathological) amino acid substitution in the mutant proteins, MAF – minor allele frequency.

## Data Availability

The datasets and materials used and/or analyzed during the current study are presented in the main paper and additional files.

## Supplementary Materials

Supplementary Materials Supplementary materials includes a description of Methods used in the study. Figure S1: craniectomy of the scaphocephalic cadaveric head. Table S1: complete list of deleterious (pathologic) genetic variants associated with the current case of sagittal CS.
