# Supplementary material for: Sagittal Craniosynostosis with Uncommon Anatomical Pathologies in a 56-Year-Old Male Cadaver
Source: Case Rep Pathol. 2019 Dec 8;2019:8034021. doi: 10.1155/2019/8034021 (PMC6925784; doi:10.1155/2019/8034021)
Supplement: Supplementary Materials — includes a description of Methods used in the study. Figure S1: craniectomy of the scaphocephalic cadaveric head. Table S1: complete list of deleterious (pathologic) genetic variants associated with the current case of sagittal CS. [file 8034021.f1.pdf]

Supplementary materials

Sagittal Craniosynostosis with Uncommon Anatomical Pathologies in a 56-Year-Old Male Cadaver

Andrey Frolov, Craig Lawson, Joshua Olatunde, James T. Goodrich, and John R. Martin, III

Methods

CT Imaging

The embalmed cadaveric head specimen underwent a CT scanning at SLU Hospital using Siemens SOMATOM Definition Flash system (140 kV; 119 mAs; slice thickness: 3mm by 3mm interval; detector size: 0.6 mm, 128 rows of detectors, pitch of 0.6). The images were obtained with standard resolution and analyzed using Syngo Fast-View software.

Measurement of Skull Bone Thickness

The thickness of mesocephalic skull bones was determined exactly as described in [1] using a digital caliper. The respective male skulls were purchased from Osta International (White Rock, Canada). The scaphocephalic bone thickness was derived from the respective calibrated CT images of the embalmed scaphocephalic head at the anatomical points matching those of mesocephalic skulls. The direct measurement of bone thickness in the scaphocephalic head which would require its maceration was not performed due to the specimen preservation for additional anatomical studies.

Anatomical Dissection

Craniectomy

A craniectomy was conducted to examine the extent of the scaphocephaly in the intracranial space. The dissection of the specimen was performed at Saint Louis University School of Medicine's Practical Anatomy and Surgical Education Learning Center at the 27th Annual Craniofacial Surgery and Transfacial Approaches to the Skull Base workshop by James T. Goodrich, M.D., Ph.D., D.Sc. (Honoris Causis) (Albert Einstein College of Medicine, New York, NY). The specimen was thawed at room temperature for 48 hours prior to dissection. The head was shaved using a razor to make more visible the surgical scars from the initial craniotomy. The scar along the midsagittal plane of the scalp highlighted with a blue marker was used to outline the incision for dissection which was made along the midsagittal plane of the scalp from anterior to posterior (Fig. S1). The scalp was isolated from the calvarium with a periosteal elevator. Areas of fibrous tissue adjacent to the bone strip were outlined with blue marker and were used for the elevator insertion into the intracranial space to separate the dura from the calvarium. Following the dura removal, the calvarium was cut open with an electrical bone saw. The cuts were made from the superior portion of the frontal bone to the superior portion of the occipital bone and the lateral aspect of both parietal bones.

Mandibulotomy

Physical examination of the maxillofacial features of the cadaveric head revealed a large under bite that prompted the dissection of the mandible to probe for additional abnormalities.

The mandible was exposed by removing the soft tissue from the mental surface followed by bisection of the bone and tongue. This procedure revealed and exostotic hard palate (torus palatinus) and complete edentulism.

Histological analysis

Bone tissue samples were collected for histological analysis. Samples included abnormal bone from the synostotic calvarium, bridging bone from the areas between the synostotic and normal bone, and the exostotic hard palate. Tissue fixation, paraffin embedding, sectioning, and staining with hematoxylin & eosin were performed by Research Microscopy and Histology Core, Department of Pathology, Saint Louis University (SLU) School of Medicine according to the standardized procedures. Images were captured on Olympus 41BX-EPI microscope equipped with the 2.5x, 5x, 10x, 20x, and 40x objectives. The data acquisition and image analysis were performed by using CellSens Standard software.

Genetic Analysis

The Next Generation Sequencing (NGS) and bioinformatics analysis were performed as previously described [2, 3] with the following modifications. DNA was extracted from the left sternocleidomastoid muscle specimen procured from the embalmed cadaveric head and was sequenced to 30x depth of coverage (~4.5 Gb) on the Illumina HiSeq 2500 NGS platform. The 30x depth of coverage fulfills a requirement for the detection of human genome mutations (10x to 30x, Illumina). DNA extraction and exome sequencing were conducted by Omega Bioservices (Norcross, GA). The cumulative exome coverage for > 10x depth of coverage was 82 % indicating that the majority of the exome was probed. The variant call and annotation were performed by Genome Technology Access Center (GTAC, Washington University in St. Louis) using SnpSift varType and ANNOVAR. The resultant data were converted into the Microsoft Excel format and pathologic/deleterious variants were identified through the consecutive filtering steps outlined in [3]. Functional annotation of the remaining variants was performed using UniProtKB Protein, Google Scholar, and PubMed database searches.

References

1. Mahinda, H.A.M. and O.P. Murty, *Variability in thickness of human skull bones and sternum - an autopsy experience.* J Forens Med Toxicol, 2009. **26**(2): p. 26-31.
2. Frolov, A., Tan, Y., Rana, M., and Martin, J.R III, *A rare case of human diphallia associated with hypospadias.* Case Rep Urol, 2018. **2018**: p. Article ID 8293036, 6 pages, https://doi.org/10.1155/2018/8293036.
3. Jenkins, M., et al., Situs inversus totalis in a 96-year-old female cadaver: evidence pointing toward the two-cilia model. It. J. Anat. Embryol. vol. 124, no. 2, pp. 230-246, 2019.

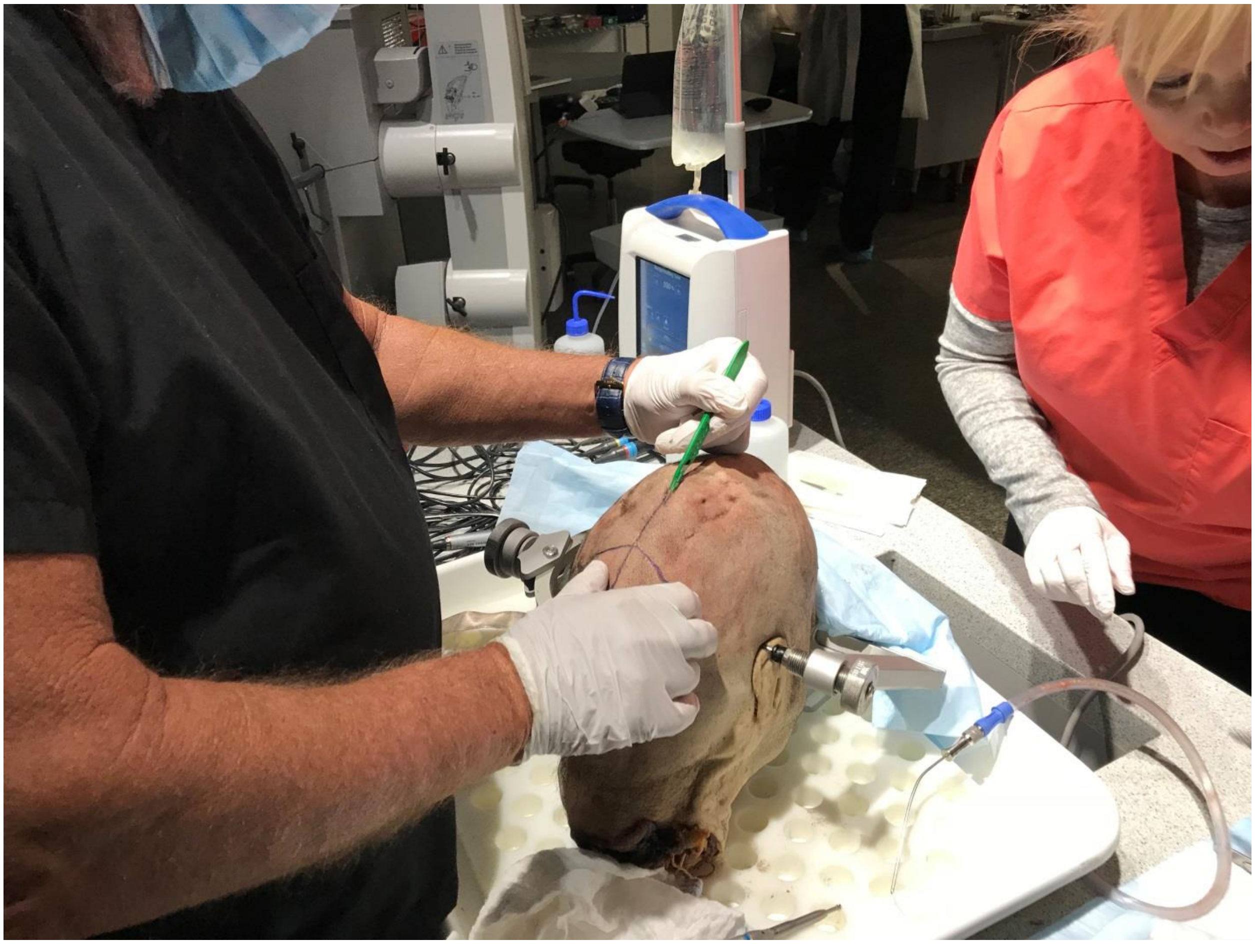

Figure S1. Craniectomy of the scaphocephalic cadaveric head. An incision was made along the midsagittal plane of the scalp from anterior to posterior.

Table S1. Complete list of deleterious (pathologic) genetic variants associated with the current case of sagittal CS.

Gene	Protein Function
ABCD1	ATP Binding Cassette Subfamily D Member 1. Probable transporter. The nucleotide-binding fold acts as an ATP-binding subunit with ATPase activity. This peroxisomal membrane protein is likely involved in the peroxisomal transport or catabolism of very long chain fatty acids. Defects in this gene have been identified as the underlying cause of adrenoleukodystrophy, an X-chromosome recessively inherited demyelinating disorder of the nervous system.
ANKRD30B	Ankyrin repeat domain-containing protein 30B.
ARHGAP21	Rho GTPase Activating Protein 21. Functions as a GTPase-activating protein (GAP) for RHOA and CDC42.
BMP6	Bone morphogenetic protein 6. Teeth development. Cartilage development. Endochondral ossification. Positive regulation of osteoblast differentiation. Positive regulation of bone mineralization. Positive regulation of chondrocyte differentiation.
C1orf106	C1orf106 chromosome 1 open reading frame 106.
CACNA1A	Voltage-dependent P/Q-type calcium channel subunit alpha-1A. Voltage-sensitive calcium channels (VSCC) mediate the entry of calcium ions into excitable cells and are also involved in a variety of calcium-dependent processes, including muscle contraction, hormone or neurotransmitter release, gene expression, cell motility, cell division and cell death.
CD101	Immunoglobulin superfamily member 2. Plays a role as inhibitor of T-cells proliferation induced by CD3. Inhibits expression of IL2RA on activated T-cells and secretion of IL2. Inhibits tyrosine kinases that are required for IL2 production and cellular proliferation. Inhibits phospholipase C-gamma-1/PLCG1 phosphorylation and subsequent CD3-induced changes in intracellular free calcium.
CDC27	Cell Division Cycle 27. Component of the anaphase promoting complex/cyclosome (APC/C), a cell cycle-regulated E3 ubiquitin ligase that controls progression through mitosis and the G1 phase of the cell cycle.
CEP162	Centrosomal protein of 162 kDa. Required to promote assembly of the transition zone in primary cilia. Acts by specifically recognizing and binding the axonemal microtubule. Required to mediate CEP290 association with microtubules.
CHRNA7	Neuronal acetylcholine receptor subunit alpha-7. After binding acetylcholine, the AChR responds by an extensive change in conformation that affects all subunits and leads to opening of an ion-conducting channel across the plasma membrane. Calcium ion transport. Positive regulation of cell proliferation.
CNN2	Calponin-2. Thin filament-associated protein that is implicated in the regulation and modulation of smooth muscle contraction. It is capable of binding to actin, calmodulin, troponin C and tropomyosin.
COL11A2	Collagen alpha2 (XI). Extracellular matrix structural constituent.
COL3A1	Collagen alpha-1(III) chain. Collagen type III occurs in most soft connective tissues along with type I collagen. Involved in regulation of cortical development. It Is the major ligand of ADGRG1 in the developing brain and binding to ADGRG1 inhibits neuronal migration and activates the RhoA pathway by coupling ADGRG1 to GNA13 and possibly GNA12.

CROCC	Rootletin. Major structural component of the ciliary rootlet, a cytoskeletal-like structure in ciliated cells which originates from the basal body at the proximal end of a cilium and extends proximally toward the cell nucleus (By similarity). Required for the correct positioning of the cilium basal body relative to the cell nucleus, to allow for ciliogenesis.
CRYGD	Gamma-crystallin D. Crystallins are the dominant structural components of the vertebrate eye lens.
CYP27A1	Sterol 26-hydroxylase, mitochondrial. Catalyzes the first step in the oxidation of the side chain of sterol intermediates; the 27-hydroxylation of 5-beta-cholestane-3-alpha,7-alpha,12-alpha-triol.
CYP4F3	Docosahexaenoic acid omega-hydroxylase CYP4F3. Isoform CYP4F3A: Catalyzes the omega-hydroxylation of leukotriene-B4, a potent chemoattractant for polymorphonuclear leukocytes, it has low activity for arachidonic acid. Isoform CYP4F3B: Shows arachidonic acid omega-hydroxylase activity by mediating conversion of arachidonic acid to 20-hydroxyeicosatetraenoic acid (20-HETE). Has a 30-fold higher Km for leukotriene-B4 compared with CYP4F3A.
CYP4V2	Cytochrome P450 4V2. Omega-hydroxylase that oxidizes medium-chain saturated fatty acids and polyunsaturated omega-3 fatty acids, and which plays a role in fatty acid and steroid metabolism in the eye.
DCAF16	DDB1- and CUL4-associated factor 16. This protein is involved in the pathway protein ubiquitination, which is part of protein modification. May function as a substrate receptor for CUL4-DDB1 E3 ubiquitin-protein ligase complex.
DDX54	ATP-dependent RNA helicase DDX54. Has RNA-dependent ATPase activity. Represses the transcriptional activity of nuclear receptors.
DENND2A	DENN domain-containing protein 2A. Guanine nucleotide exchange factor (GEF) which may activate RAB9A and RAB9B. Promotes the exchange of GDP to GTP, converting inactive GDP-bound Rab proteins into their active GTP-bound form. May play a role in late endosomes back to trans-Golgi network/TGN transport.
DNAH11	Dynein heavy chain 11, axonemal. Force generating protein of respiratory cilia. Produces force towards the minus ends of microtubules. Dynein has ATPase activity; the force-producing power stroke is thought to occur on release of ADP.
EDEM3	ER degradation-enhancing alpha-mannosidase-like protein 3. Involved in endoplasmic reticulum-associated degradation (ERAD). Accelerates the glycoprotein ERAD by proteasomes, by catalyzing mannose trimming from Man8GlcNAc2 to Man7GlcNAc2 in the N-glycans.
EIF4E1B	Eukaryotic translation initiation factor 4E type 1B. Recognizes and binds the 7-methylguanosine-containing mRNA cap during an early step in the initiation of protein synthesis and facilitates ribosome binding by inducing the unwinding of the mRNAs secondary structure.
ENDOG	Endonuclease G, mitochondrial. Cleaves DNA at double-stranded (DG)n.(DC)n and at single-stranded (DC)n tracts. In addition to deoxyribonuclease activities, also has ribonuclease (RNase) and RNase H activities. Capable of generating the RNA primers required by DNA polymerase gamma to initiate replication of mitochondrial DNA (By similarity).
EXD3	Exonuclease mut-7 homolog. Possesses 3'-5' exoribonuclease activity. Required for 3'-end trimming of AGO1-bound miRNAs (By similarity).
FAM187B	Protein FAM187B. Transmembrane protein 162.

FANCC	Fanconi anemia group C protein. DNA repair protein that may operate in a post-replication repair or a cell cycle checkpoint function. May be implicated in interstrand DNA cross-link repair and in the maintenance of normal chromosome stability. Upon IFNG induction, may facilitate STAT1 activation by recruiting STAT1 to IFNGR1.
FPR2	N-formyl peptide receptor 2. Low affinity receptor for N-formyl-methionyl peptides, which are powerful neutrophils chemotactic factors. Binding of FMLP to the receptor causes activation of neutrophils. This response is mediated via a G-protein that activates a phosphatidylinositol-calcium second messenger system. The activation of LXA4R could result in an anti-inflammatory outcome counter-acting the actions of proinflammatory signals such as LTB4 (leukotriene B4).
FRG2B	Protein FRG2-like-1.
GBP6	Guanylate-binding protein 6. Binds GTP, GDP and GMP.
GMIP	GEM-interacting protein. Stimulates, in vitro and in vivo, the GTPase activity of RhoA.
GRAMD1A	GRAM domain-containing protein 1A. May play a role in tumor progression.
GUCY2F	Retinal guanylyl cyclase 2. Probably plays a specific functional role in the rods and/or cones of photoreceptors. It may be the enzyme involved in the re-synthesis of cGMP required for recovery of the dark state after phototransduction.
HAS3	Hyaluronan synthase 3. Catalyzes the addition of GlcNAc or GlcUA monosaccharides to the nascent hyaluronan polymer. Therefore, it is essential to hyaluronan synthesis a major component of most extracellular matrices that has a structural role in tissues architectures and regulates cell adhesion, migration and differentiation.
IGF2R	Cation-independent mannose-6-phosphate receptor. Transport of phosphorylated lysosomal enzymes from the Golgi complex and the cell surface to lysosomes. This receptor also binds IGF2. Acts as a positive regulator of T-cell co-activation, by binding DPP4.
INADL	InaD-like protein also known as PATJ. Negative regulator of Wnt signaling. Blocks DFz1 activity in the planar cell polarity pathway (PCP) in cooperation with atypical PKC. Fzd/PCP pathway represents the non-canonical Wnt signaling.
KLHL30	Kelch-like protein 30. Protein ubiquitination.
MAN2B2	Epididymis-specific alpha-mannosidase. Mannose metabolic process. Protein deglycosylation.
MAP7D3	MAP7 domain-containing protein 3. Promotes the assembly and stability of microtubules.
MED1	Mediator of RNA polymerase II transcription subunit 1. Component of the Mediator complex, a coactivator involved in the regulated transcription of nearly all RNA polymerase II-dependent genes. Ligand-dependent nuclear receptor binding. Ligand-dependent nuclear receptor transcription coactivator activity.
MFGE8	Lactadherin. Plays an important role in the maintenance of intestinal epithelial homeostasis and the promotion of mucosal healing. Promotes VEGF-dependent neovascularization (By similarity).
MPZL2	Myelin protein zero-like protein 2. Mediates homophilic cell-cell adhesion.
MROH2B	Maestro heat-like repeat-containing protein family member 2B. May play a role in the process of sperm capacitation.

MST1	Hepatocyte growth factor-like protein. Negative regulation of gluconeogenesis.
MST1L	Putative macrophage stimulating 1-like protein.
MTCH2	Mitochondrial carrier homolog 2. The substrate transported is not yet known. Induces mitochondrial depolarization.
MUC5B	Mucin-5B. Gel-forming mucin that is thought to contribute to the lubricating and viscoelastic properties of whole saliva and cervical mucus.
MYO15A	Unconventional myosin-XV. Myosins are actin-based motor molecules with ATPase activity. Unconventional myosins serve in intracellular movements.
NCOR2	Nuclear receptor corepressor 2. Transcriptional corepressor. Mediates the transcriptional repression activity of some nuclear receptors by promoting chromatin condensation, thus preventing access of the basal transcription.
NDOR1	NADPH-dependent diflavin oxidoreductase 1. Component of the cytosolic iron-sulfur (Fe-S) protein assembly (CIA) machinery. Required for the maturation of extramitochondrial Fe-S proteins (By similarity).
NLRP7	NACHT, LRR and PYD domains-containing protein 7. Inhibits CASP1/caspase-1-dependent IL1B secretion. Embryonic development.
NOS3	Nitric oxide synthase, endothelial. Produces nitric oxide (NO) which is implicated in vascular smooth muscle relaxation through a cGMP-mediated signal transduction pathway.
NUMBL	Numb-like protein. Plays a role in the process of neurogenesis. Required throughout embryonic neurogenesis to maintain neural progenitor cells, also called radial glial cells (RGCs), by allowing their daughter cells to choose progenitor over neuronal cell fate. Negative regulator of NF-kappa-B signaling pathway.
OR13C5	Olfactory receptor 13C5. Odorant receptor.
OR5J2	Olfactory receptor 5J2, Odorant receptor.
PARD3B	Partitioning defective 3 homolog B. Putative adapter protein involved in asymmetrical cell division and cell polarization processes. May play a role in the formation of epithelial tight junctions.
PCDHB3	Protocadherin beta-3. Potential calcium-dependent cell-adhesion protein. May be involved in the establishment and maintenance of specific neuronal connections in the brain.
PCNXL2	Pecanex-like protein 2. May play a role in tumorigenesis of colorectal carcinomas with high microsatellite instability (MSI-H).
PIEZO1	Piezo-type mechanosensitive ion channel component 1. Pore-forming subunit of a mechanosensitive non-specific cation channel. Plays a key role in osteogenesis. Its activation commits mesenchymal stem cells to osteogenic differentiation.
PIP	Prolactin-inducible protein. Negative regulation of T cell apoptotic process. Positive regulation of gene expression. Detection of chemical stimulus involved in sensory perception of bitter taste.
PLBD1	Phospholipase B-like 1. In view of the small size of the putative binding pocket, it has been proposed that it may act as an amidase or a peptidase (By similarity).
PSMB11	Proteasome subunit beta type-11. The proteasome is a multicatalytic proteinase complex which is characterized by its ability to cleave peptides with Arg, Phe, Tyr, Leu, and Glu adjacent to the leaving group at neutral or slightly basic pH.

RASAL1	RasGAP-activating-like protein 1. Probable inhibitory regulator of the Ras-cyclic AMP pathway. Plays a role in dendrite formation by melanocytes.
REC8	Meiotic recombination protein REC8 homolog. Required during meiosis for separation of sister chromatids and homologous chromosomes.
RNF213	E3 ubiquitin-protein ligase RNF213. Involved in the non-canonical Wnt signaling pathway in vascular development: acts by mediating ubiquitination and degradation of FLNA and NFATC2 downstream of RSPO3, leading inhibition of the non-canonical Wnt signaling pathway and promoting vessel regression.
RPP40	Ribonuclease P protein subunit p40. Component of ribonuclease P, a protein complex that generates mature tRNA molecules by cleaving their 5'-ends.
SCARF2	Scavenger receptor class F member 2. Probable adhesion protein, which mediates homophilic and heterophilic interactions. In contrast to SCARF1, it poorly mediates the binding and degradation of acetylated low density lipoprotein (Ac-LDL)
SYT15	Synaptotagmin-15. May be involved in the trafficking and exocytosis of secretory vesicles in non-neuronal tissues.
TBC1D2	TBC1 domain family member 2A. Acts as GTPase-activating protein for RAB7A. Signal effector acting as a linker between RAC1 and RAB7A, leading to RAB7A inactivation and subsequent inhibition of cadherin degradation and reduced cell-cell adhesion.
TBXAS1	Thromboxane-A synthase. Oxidoreductase activity, acting on paired donors, with incorporation or reduction of molecular oxygen. Heme binding. Iron ion binding.
TMEM255B	Transmembrane protein 255B.
TMEM41A	Transmembrane protein 41A.
TNS4	Tensin-4. May be involved in cell migration, cartilage development and in linking signal transduction pathways to the cytoskeleton (By similarity). May promote apoptosis, via its cleavage by caspase-3.
XIRP1	Xin actin-binding repeat-containing protein 1. Protects actin filaments from depolymerization.
ZNF563	Zinc finger protein 563. Regulation of transcription, DNA-templated.
ZNF597	Zinc finger protein 597. Regulation of transcription, DNA-templated.
ZP3	Zona pellucida sperm-binding protein 3. The mammalian zona pellucida, which mediates species-specific sperm binding, induction of the acrosome reaction and prevents post-fertilization polyspermy, is composed of three to four glycoproteins, ZP1, ZP2, ZP3, and ZP4. ZP3 is essential for sperm binding and zona matrix formation.
